# Pulverized Sewage Sludge Combustion with Potassium Chloride Addition—Fate of Phosphorus, Potassium, Sulfur, and Heavy Metals

**DOI:** 10.1021/acsomega.5c01774

**Published:** 2025-06-11

**Authors:** Andreas Ewald, Gabriel Roeder, Hartmut Spliethoff, Sebastian Fendt

**Affiliations:** Technical University of Munich, Germany; TUM School of Engineering and Design, Department of Mechanical Engineering, 9184 Chair of Energy Systems, Boltzmannstraße 15, Garching 85748, Germany

## Abstract

Sewage sludge is a sink for heavy metals and plant nutrients. Thermal treatment of sewage sludge can remove heavy metals, providing a potential raw material for fertilizer production. Stationary fluidized bed combustion is a common thermal treatment for sewage sludge, with its process-related maximum temperature of 850 °C. This temperature sometimes does not sufficiently remove heavy metals. As an alternative, we demonstrated pulverized combustion in an entrained flow reactor at 850 and 1100 °C, including the addition of KCl. Thermodynamic equilibrium calculations allowed for the assessment of reaction conditions and the effect of additives on the evaporation of heavy metals. Pulverized combustion at 850 °C enabled the partial removal of Hg, Tl, Pb, As, and Zn from the bottom ash. Pulverized combustion at 1100 °C combined with the addition of KCl evaporated 51% of Cu, 55% of Zn, 60% of As, 70% of Pb, 72% of Cd, 83% of Tl, and 91% of Hg. Due to the enhanced evaporation of Cd and Cu, this ash complied with all heavy metal limit values of the German Fertilizer Ordinance, except for Ni. Overall, subsequent ash separation at high temperatures enabled the valorization of sewage sludge contaminated with heavy metals. P and K remained in the ash, showing the potential of this thermal treatment method for nutrient recovery.

## Graphical abstract

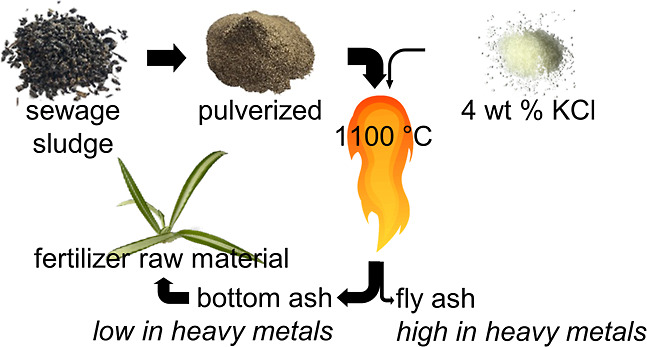

## 1 Introduction

Due to its high content of inorganic substances, sewage sludge contains valuable plant nutrients like P, making it a suitable organic fertilizer when properly stabilized.^1,2^ However, possible contamination with organic pollutants, such as pharmaceutical residues, and heavy metals limits its agricultural use.^3^ Sewage sludge used as a fertilizer can also introduce viruses^4^ and microplastics into the environment,^5^ which necessitates continuous monitoring.^3^

Thermal treatment can destroy organic pollutants or remove inorganic contaminants,^6,7^ allowing the resulting ash to serve as a raw material for nutrient recovery.^6–9^ Some methods also generate usable energy.^6,10^ In Germany, thermal treatment has become increasingly important,^7,11,12^ with a growing share from 10% in 1991 to over 70% in 2017. Established methods include coincineration and monoincineration, alongside alternative methods like gasification and pyrolysis.^11^

The German Ordinance on the Utilization of Sewage Sludge, Sewage Sludge Mixture and Sewage Sludge Compost (AbfKlärV) now mandates P recovery in sewage sludge disposal, allowing P to be recycled from either sewage sludge or its ash. In the future, no other types of ash may cause the dilution of P in sewage sludge ash (SSA). Thus, AbfKlärV permits coincineration only with coal or gas, excluding other residuals to support the thermal treatment.^13^

This ordinance favors monoincineration of sewage sludge, with the stationary fluidized bed combustion being the preferred method.^7,11,12^ The Ordinance on the Incineration and Coincineration of Waste (17th BImSchV) sets process parameters, generally aligning with the EU Industrial Emissions Directive 2010/75/EU. To crack organic pollutants, a minimum exhaust gas temperature of 850 °C and a minimum residence time of 2 s are required after the final air injection.^14^ However, stationary fluidized bed combustion cannot safely reach a significant higher temperature,^11,15^ as SSA tends to sinter and agglomerate,^11,16^ with the maximum temperature limit for this method being around 950 °C.^11^ This moderate combustion temperature concentrates the P in the ash, facilitating its subsequent recovery.^7,12^

A challenge in recovering P from SSA is its contamination with heavy metals.^7,17^ In Germany, the Fertilizer Ordinance (DüMV) sets limits for specific heavy metals.^18^ Testing samples from 24 of 26 monoincineration facilities in Germany, Herzel et al. found that 13 exceeded heavy metal limit values from DüMV, requiring further processing for P recovery.^7^ However, combustion parameters can reduce heavy metals in SSA.^15^ For example, volatile metals evaporate depending on combustion temperature and accumulate downstream in the fly ash, while less volatile metals remain in the bottom ash.^19–21^ Increasing treatment temperatures from 850 to 1100 °C has a positive effect on the evaporation of most heavy metals.^15,22–24^ While fluidized bed combustion operates in a temperature range of up to 900 °C, the typical temperature range of grate firing and suspension firing is above 1000 °C.^25^

Even though the AbfKlärV only permits coincineration with coal or gas,^13^ there are studies on how other substrates affect heavy metals in thermal treatment of sewage sludge.^26–28^ A common alternative is the addition of Cl via additives to reduce heavy metals in SSA.^15,20,29–32^ When adding 13 different additives before combustion, only the five chlorides showed significant heavy metal reduction compared to the control sample.^33^ Cr, Cu, Ni, Pb, and Zn tend to form volatile chlorides.^30,34^ At about 1000 °C, Cd, Zn, Pb, Cu, Ni, and Cr chlorides are largely in the vapor phase due to their vapor pressure. The respective heavy metal oxides have vapor pressures several orders of magnitude lower.^29^ The following mechanism for the chlorination of heavy metals is suggested in the literature. A Cl donor can react with a metal oxide in two possible ways. If there is direct contact between the two reactants, direct chlorination can occur according to eq 1. Indirect chlorination can occur according to eqs 2 and 3 or 4 and 5, in which the overall reaction is divided into two phases.^35–40^ Fraissler et al. showed through thermodynamic equilibrium calculations (TEC) that relevant factors in real mixtures can be more diverse and that various components influence the evaporation of heavy metals.^30^ Cl introduced as MgCl_2_ or NaCl reduces heavy metals more effectively than organically bound Cl.^41,42^ Commonly used salts for adding Cl in the thermal treatment of sewage sludge are MgCl_2_,^15,29,33,37–39,41,43–48^ CaCl_2_,^20,29,30,33,37,38,40,44,46–49^ NaCl,^32,33,37,38,42,44,48,50^ and KCl.^37,39,43,48^ Additionally, adding MgCl_2_ or KCl improves P retention during sewage sludge incineration,^39^ and adding KCl at a K to P molar ratio of 2.5 increases P bioavailability.^7^

1 MO+XCl→MCl+XO

2 $$2XCl+O_{2}\to 2XO+{Cl}_{2}$$

3 $$2MO+{Cl}_{2}\to 2MCl+O_{2}$$

4 $$XCl+H_{2}O\to XO+2HCl$$

5 $$MO+2HCl\to MCl+H_{2}O$$

Pulverized fuel combustion is a versatile technology with high energy densities in the combustion chamber and generally low residual oxygen levels in the flue gas. Therefore, very high temperatures are achievable. Due to the low residence times of the solid particles in the hot zone, the load of the boilers with pulverized fuel combustion can be controlled with ease and undergo fast alterations. A disadvantage of this technology is the high degree of fuel preparation needed with constant quality.^51–53^ The combustion of biomass, especially at higher temperatures, as in the case of pulverized fuel combustion, can add further drawbacks with fuels containing a high amount of inorganic compounds. These compounds can contribute to slagging, the corrosion of the heat exchange surfaces, and increase the formation of aerosols.^54–56^ Additionally, higher combustion temperatures can contribute to a higher NO_*x*_ content in the flue gas.^57,58^

This study investigated pulverized sewage sludge combustion (PSSC) and proposes a potential alternative to stationary fluidized bed combustion for sewage sludge incineration, enabling higher temperatures^51–53^ for a potentially more effective heavy metal evaporation from SSA. The experimental setup used allowed for comparable conditions to full-scale furnaces. In addition, the improvement of heavy metal evaporation using an additive was investigated. TEC with FactSage can predict the heavy metal removal during thermal treatment, including the effect of adding chlorine-containing additives.^30,33^ TEC of the sewage sludge used when adding KCl, MgCl_2_, or CaCl_2_ predicted increased evaporation for some elements. Experimental combustion of sewage sludge at 850 and 1100 °C showed the significance of temperature and whether the shorter residence time of PSSC was sufficient to remove heavy metals from the ash and which heavy metals were affected. For reliable processing in experimental combustion, the sewage sludge was torrefied and KCl was chosen as an additive. The goal was a SSA rich in plant nutrients and low in heavy metals considering the following elements. The primary plant nutrients in SSA are P and K.^2^ S leads to corrosion during combustion and causes environmental damage when released with the exhaust gas.^16^ All inorganic pollutants and heavy metals (As, Cd, Cu, Cr, Hg, Ni, Pb, Tl, Zn) with labeling thresholds and limit values in DüMV from Table 1 were part of the evaluation.^18^ The concept relies on the implementation of cyclone separation or filtration at high temperatures. It should be noted that the efficiency of cyclone separation decreases as the temperature increases.^59,60^ For hot-gas filtration, high-temperature steel can be used up to 650 °C and with ceramic filter media, a temperature up to 1000 °C and higher is possible.^61^

**Table 1:** Labeling Thresholds and Limit Values according to DüMV^62^

element content (mg/kg)	labeling threshold	limit values
arsenic (As)	20	40
cadmium (Cd^a^)	1.0	1.5
copper (Cu)	500	900
chromium (Cr)	300	
mercury (Hg)	0.5	1.0
nickel (Ni)	40	80
lead (Pb)	100	150
thallium (Tl)	0.5	1.0
zinc (Zn)	1000	4000

a Only when the P_2_O_5_ content exceeds 5 wt %.

In the research project PyroGasII (FKZ: 03EI5457A), the torrefied sewage sludge (tSS) used was gasified in an industry-like entrained flow reactor.^63^ This study allows a comparison of gasification and combustion of the same fuel.

The ongoing research project DreiSATS (FKZ: 02WPR1544) under the RePhoR (Regional P Recycling) initiative investigates a similar approach, showing the interest in pulverized fuel combustion for sewage sludge treatment.^64^ Here, sewage sludge is incinerated in a dust-firing process. A special impulse burner and the addition of tertiary air avoid maintenance-intensive refractory lining of the combustion chamber. Hot gas filtration separates the ash at around 800 °C, before the heat from the flue gas is used. Adding additives before combustion modifies the SSA to reduce heavy metals and improve phosphate solubility. A fertilizer is created from the SSA through subsequent acid leaching and granulation steps.^65^

To the best of our knowledge, no results from PSSC with additives have been published so far. Sewage sludge incineration with increased combustion temperature represents an innovative method to generate SSA, which can be further used with regard to P recovery. In Germany, the obligation to recover P according to the AbfKlärV will come into force in 2029 for large wastewater treatment plants and in 2032 for smaller wastewater treatment plants,^13^ which highlights the need to improve current processes.

## 2 Experimental Section

### 2.1 Combustion System and Reaction Conditions

To generate ash, a muffle furnace (MF) from Nabertherm GmbH and the Entrained Flow Combustion Reactor (EFCoRe) were used. Figure 1 provides a schematic representation of the combustion reactor. The electric heating of the reactor allowed for continuous temperature control. For this study, the fuel input was between 4 and 5 kW, with a target air-fuel equivalence ratio λ of 1.15. This air-fuel equivalence ratio has been used for this type of reactor and yields low excess oxygen in the flue gas and a good fuel burn-out.^57,66^ A swirl burner introduced the powdered sewage sludge from the top of the reactor, along with air. This burner had both a primary and a secondary airflow. The primary airflow acted as the carrier gas. The secondary airflow surrounded the fuel in a concentric tube, causing the air-fuel mixture to swirl within the reactor. The primary to secondary air ratio was one part primary air to two parts secondary air. The sewage sludge reacts as it travels downward through a ceramic tube. The gas residence time in the hot zone was around 5 s.

**Figure 1:**
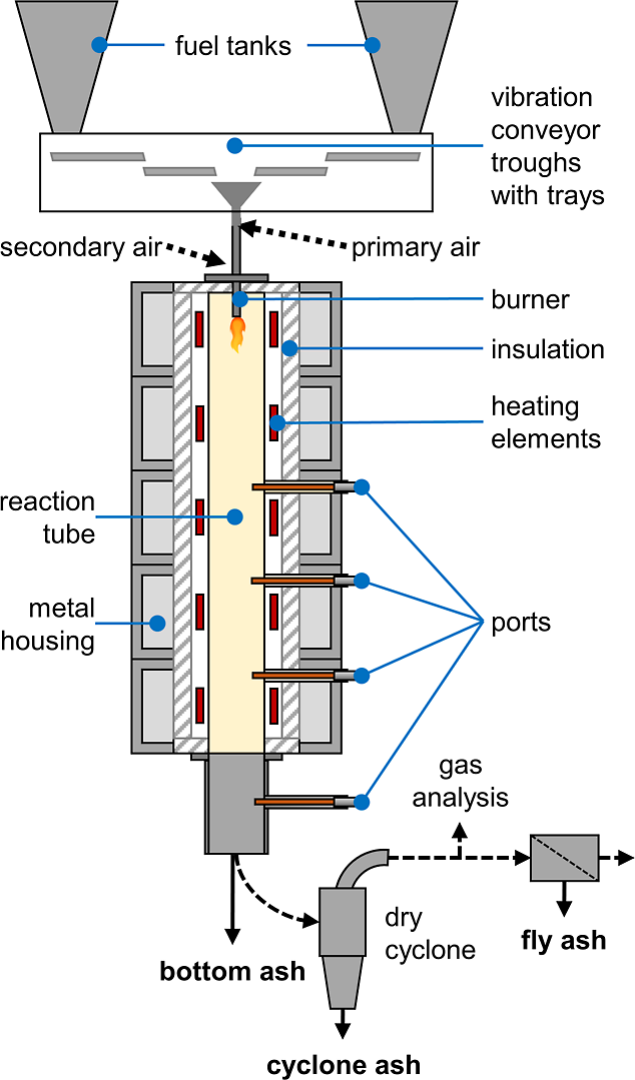
Simplified flowsheet of the Entrained Flow Combustion Reactor (EFCoRe) with ash sampling locations.

After the reaction tube, the SSA and flue gases entered a convective section. This section included an ash container at the base of the reactor, collecting the bottom ash. A subsequent dry cyclone separated particles up to 3 μm as the cyclone ash, and a dust filter collected the fly ash. Since the ash left the reactor with some losses in dead zones, we could not establish a reliable mass balance. The EFCoRe divided the SSA primarily into bottom ash and cyclone ash, with a comparatively small amount of fly ash. A fan maintained a constant negative relative pressure of 20 mbar within the reactor.

Side openings, or ports, allowed for gas sampling and the injection of tertiary air or reducing agents like ammonia. Near the end of the reactor, at the third port level, a high-temperature air-cooled sampling lance with a ceramic tip extracted flue gas samples. The flue gas passed through an electrically heated FSS-H350 filter by M&C TechGroup Germany GmbH at 350 °C. High-temperature filtering prevented ammonia loss due to adsorption on solids or salt formation and protects the gas measurement equipment.

An ABB AO2020 gas analyzer detected gas components, including CO, CO_2_, O_2_, NO, NO_2_, and SO_2_. At each configuration of process parameters, combustion was maintained for at least 10 min to achieve a stable and valid measurement despite minor fluctuations. Detected gas components were converted to 11% O_2_ to exclude potential dilution with ambient or excess air.

The maximum combustion temperature of the reactor is 1550 °C due to the electrical heating elements. However, the reactor was not designed for a slagging operation. Excessive slag could clog the ports or damage the reaction tube. Therefore, the maximum combustion temperature also depends on the ash melting temperature of the fuel used.

### 2.2 Methodology and Calculations

Fuel input *P*_fuel_ was set using eq 6 with the mass flow of the fuel ${ṁ}_{fuel}$ and its lower heating value *H*_i,__fuel_. The lower heating value of the fuel and therefore the fuel input differed with and without KCl.

$$P_{fuel}={ṁ}_{fuel}H_{i,fuel}$$ 6

Equation 7 defines the air-fuel equivalence ratio λ as a quotient of the volume flow of air ${\mathrm{V̇}}_{air}$ and the stoichiometric volume flow of O_2_ for a complete combustion ${\mathrm{V̇}}_{O_{2},stoic}$ . The volume flow of air ${\mathrm{V̇}}_{air}$ multiplied by 0.21 equals the volume flow of pure O_2_.

7 $$\lambda =\frac{0.21{\mathrm{V̇}}_{air}}{{\mathrm{V̇}}_{O_{2},stoic}}$$

To exclude any potential air dilution in emission tracking, measured gas concentrations can be converted based on a reference O_2_ level. Equation 8 defines the concentration of an emission at reference O_2_ *c*_*x*,ref_ with the measured concentration of the emission *c*_*x*,ref_ and the measured O_2_ level $y_{O_{2},m}$ . The 17th BImSchV regulates the thermal treatment of waste, including sewage sludge and specifies a reference O_2_ level $y_{O_{2},ref}$ of 0.11.

8 $$c_{x,ref}=\frac{{0.21-y}_{O_{2},ref}}{{0.21-y}_{O_{2},m}}\times c_{x,m}$$

For evaluating the performance of the combustion, the ash content according to DIN 51719 at 815 °C acted as a tracer, assuming the same amount of ash present before and after combustion. Tremel et al. described this ash tracer method in detail.^67^ According to eq 9, the overall conversion of the organic matter *X*_ov_ is determined, revealing an incomplete combustion. The organic matter is the sum of the volatile matter and the fixed carbon content. The ash content in the fuel *x*_ash,0_ and the ash content after the combustion ash *x*_ash_ are taken into account.

9 $$X_{ov}=\frac{1-x_{ash,0}/x_{ash}}{1-x_{ash,0}}$$

To estimate the evaporation of different elements, the ash content can cause a significant distortion. Volatile elements in the ash falsify the result from the ash tracer method. Liaw et al. receive different levels of inaccuracy when looking at different single elements as an alternative tracer.^68^ Based on their recommendations and our experience, we selected iron as a tracer to estimate the evaporation of different elements. The iron content was determined with good reproducibility and the sewage sludge contained a high iron content, making the results more reliable. Equation 10 calculates the iron tracer content for a specific element *x*_*i*,Fe_, with the iron content in the fuel *x*_Fe,0_ and in the ash *x*_Fe_

10 $$x_{i,Fe}=\frac{x_{i,0}x_{Fe}}{x_{Fe,0}}$$

TEC in FactSage 8.1 of tSS with and without an additive estimated the evaporation in advance. The Equilib module with Gibbs energy minimization enabled the calculation of complex heterogeneous equilibriums. Only gaseous and solid phases and no liquid phases were taken into account. The databases FactPS and GTOX provided the necessary information. In the case of duplicates, the data from GTOX were prioritized. The characteristics of tSS provided the data for the TEC. A gas atmosphere with O and N, corresponding to an air-fuel equivalence ratio of λ = 1.15 was added. Due to the frequent use of MgCl_2_,^15,29,33,37–39,41,43–48^ CaCl_2_,^29,30,33,37,38,40,44,46–49^ and KCl^37,39,43,48^ in literature, TEC for these salts allowed for comparison. The characteristics of tSS with added KCl used (tSS+) provided the data for KCl addition. For MgCl_2_ and CaCl_2_ addition, 4 wt % of anhydrous salt was added to the characteristics of tSS.

### 2.3 Sewage Sludge Characteristics

For reliable processing, tSS was combusted. Torrefaction is a thermal pretreatment method that can enhance solid fuels.^16^ The pretreatment reduces particle size after grinding due to fiber brittleness and improves the heating value of dried sewage sludge (dSS) with a maximum at 240–250 °C.^69,70^ dSS was torrefied at 300 °C for 20 min to obtain tSS. A Retsch SR300 rotor beater mill equipped with a 250 μm sieve ground the tSS used, to produce a free-flowing powder suitable for the EFCoRe. Thermal pretreatment made grinding of tSS easier, reducing energy consumption and resulting in a smaller average particle size with the same sieve when compared to ground dSS.

MgCl_2_ and CaCl_2_ led to a clumping powder when added as dry salt to the pulverized sewage sludge. Due to its nonhygroscopic behavior, KCl allowed dry addition without clumping. Since KCl addition in SSA can improve heavy metal evaporation^37,39,43^ and increase plant availability,^7^ 4 wt % of powdered anhydrous KCl was added by weight before grinding. Based on the ash content of tSS, this is approximately 100 g KCl/kg SSA or 48 g Cl^–^/kg SSA. A significant effect on the evaporation of heavy metals was observed in literature for this amount of Cl addition,^15,29,43^ although laboratory-scale studies also use higher Cl contents.^37,39,48^ To ensure technical feasibility, the resulting chlorine content in the fuel in this study was within the typical range for municipal waste.^71^ To ensure comparability, we used the same amount of KCl for an experimental study on heavy metal evaporation in gasification.^63^ Grinding tSS with added KCl together ensured a uniform, homogeneous mixing and resulted in tSS+.

For fuel characterization, proximate analysis and elemental analysis were conducted and heating value and ash melting behavior were determined. Table 2 shows the results of the general fuel characterization.

**Table 2:** Fuel Analysis of the Sewage Sludge^a^

	dSS	tSS	tSS+
proximate analysis (wt %)			
moisture	9.8	3.2	4.9
volatile yield	47.6	47.8	45.1
ash	39.3	41.6	43.5
fixed carbon	3.3	7.4	6.6
ultimate analysis (wt %)			
carbon (C)	27.2	29.8	29
hydrogen (H)	2.9	3.6	3.4
nitrogen (N)	3.5	4.2	4.1
sulfur (S)	0.2	0.2	0.2
chlorine (Cl)	0.1	0.1	1.8
oxygen (O)	26.6	20	17.6
heating value (MJ/kg)			
higher heating value	12.3	13.4	13.5
lower heating value	11.3	12.5	12.6
ash melting temperature (°C)			
shrinkage starting temperature	890	925	1018
deformation temperature	943	1116	1138
hemisphere temperature	1216	1203	1271
flow temperature	1241	1232	1281

a All values on an as received basis.

The proximate analysis included a moisture analysis according to DIN EN ISO 18134, a volatile matter analysis according to DIN EN ISO 18123, and a gravimetric ash analysis according to DIN EN ISO 18122 at 550 °C. The fixed carbon content was calculated. Due to pretreatment, tSS was less hygroscopic compared to dSS, with a reduced moisture content when in balance with air humidity. Ash content and fixed carbon content of tSS increased due to thermal pretreatment. Adding KCl to obtain tSS+ also raised the ash content of the fuel.

Ultimate analysis was performed using a Vario Macro Elemental Analyzer according to DIN EN ISO 16948. The Cl content came from a specialized Cl element analyzer equipped with a silver-based electrochemical detector. Adding KCl to obtain tSS+ increased the Cl content by 1.9 wt % according to a calculation, but only 1.7 wt % according to the measurement. For comparison, municipal waste typically contains 0.41% to 1.91% chlorine, with an average of 0.76%.^71^

The heating value of the solid fuel was determined according to DIN EN ISO 18125. Thermal pretreatment raised the heating value of tSS, while adding KCl only slightly influenced the heating value of tSS+.

Ash melting behavior was assessed according to DIN EN ISO 21404 with an ash melting microscope. Reproducibility for the deformation temperature was inconsistent with our equipment. However, pretreatment had little effect on the shrinkage starting temperature (SST), hemisphere temperature (HT), and flow temperature (FT) of tSS. The addition of KCl slightly increased the SST, HT, and FT of tSS+. The temperature for the investigation of PSSC was derived from the ash melting behavior. Since the reactor used only allowed for nonslagging operation, the highest possible combustion temperature was set to 1100 °C. This maintained a sufficient distance from the HT and the FT. While 1100 °C represents a typical temperature for grate or suspension firing,^25^ 850 °C was selected to cover operating conditions for fluidized bed incineration.^11,15,16,25^ An experimental study on heavy metal evaporation in gasification of the same sewage sludge performed in an industry-like entrained flow reactor reached a temperature of 1050 °C below the flame.^63^

To evaluate the fate of P, K, S, and heavy metals, the solid fuel and the three different ash samples from the EFCoRe were further characterized. Table 3 shows the content of P, K, S, other major elements, and heavy metals for dSS, tSS, tSS+. The ash content of tSS increased due to thermal pretreatment. Therefore, the content of major and trace elements in tSS increased when compared to dSS. Observed deviations are within the expected measurement accuracy. Adding KCl to obtain tSS+ significantly raised the content of K. By adding KCl, the content of heavy metals was slightly diluted.

**Table 3:** Major and Trace Elements of the Sewage Sludge^a^

	dSS	tSS	tSS+
major element composition (mg/kg)			
aluminum (Al)	^b^	31956	33849
calcium (Ca)	41328	38557	37730
iron (Fe)	26839	28310	27633
magnesium (Mg)	4357	6737	6880
phosphorus (P)	28173	29448	28838
potassium (K)	4136	5046	21522
sodium (Na)	1308	1283	1243
silicon (Si)	^b^	31305	31315
sulfur (S)	2194	2294	2135
titan (Ti)	1218	1497	1585
trace element composition (mg/kg)			
arsenic (As)	14.4	13.6	13.0
cadmium (Cd)	1.1	1.6	1.6
copper (Cu)	420	530	475
chromium (Cr)	86	74	56
mercury (Hg)	0.28	0.25	0.26
nickel (Ni)	50	47	41
lead (Pb)	49	54	47
thallium (Tl)	^b^	0.5	0.4
zinc (Zn)	1143	1310	1230

a All values on an as received basis.

b No valid data.

P, K, and S and other major elements for implementing the TEC (Al, Ca, Fe, Mg, Na, Si, Ti) were determined in the inductively coupled plasma optical emission spectroscopy according to DIN EN ISO 22022–2. Inorganic pollutants or heavy metals were considered based on labeling thresholds or limit values of the DüMV in Table 1.^18^ An accredited laboratory of the Eurofins Umwelt Ost GmbH determined As, Cd, Cu, Cr, Ni, Pb, Tl, and Zn according to DIN EN ISO 17294 by inductively coupled plasma mass spectrometry and Hg according to DIN 22022–4 by atomic absorption spectroscopy. To test the reproducibility of the results, one sample was sent to the accredited laboratory four times without declaration. The relative standard deviation for each individual heavy metal was a maximum of 6%. This uncertainty includes both the sampling and the laboratory’s measurement uncertainty.

## 3 Results and Discussion

### 3.1 Thermodynamic Equilibrium Calculations

Entering the composition of elements from tSS in FactSage 8.1 resulted in 757 possible gas phases and 1314 possible solid phases. Figure 2 shows the percentage of various elements in the gas phase across a temperature range from 0 °C to 2500 °C, in 10 °C increments, for tSS (a), tSS+ (b), tSS with added 4 wt % MgCl_2_ (c) and tSS with added 4 wt % CaCl_2_ (d).

**Figure 2:**
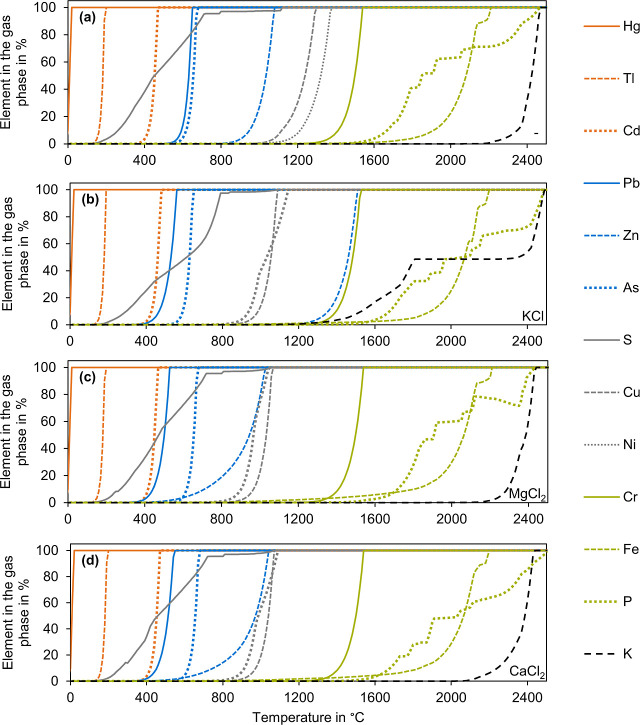
Share of different elements in the gas phase according to TEC for tSS (a), tSS+ (b), tSS with 4 wt % MgCl_2_ (c), tSS with 4 wt % CaCl_2_ (d).

With the exception of some elements like S, K, and P, most elements evaporated within a narrow temperature range in TEC. P remained in the solid phase below a temperature of 1500 °C. At a total P content below 10%, Bourgel et al. expected no significant evaporation up to 1500 °C.^72^ According to Figure 2, the evaporation of P starts at approximately 1500 °C under the conditions investigated. According to TEC, at the combustion temperature examined of at maximum 1100 °C, P remains in the SSA. Adding KCl increased the share of K in the gas phase across a broader temperature range. The evaporation began at approximately 1200 °C instead of 2200 °C, with a full evaporation of K near the same temperature.

For PSSC of tSS at λ = 1.15, the elements evaporated with increasing temperature in the following order: Hg, Tl, Cd, Pb, As, Zn, S, Cu, Ni, Cr, Fe, K, and P. Cheng et al. clustered heavy metals in incineration of sewage sludge at 850 °C into nonvolatiles (P, Fe, K, Cu, Cr, and Ni), semivolatiles (Zn, Pb, As, and Cd), and volatiles (Hg and S).^73^ According to TEC, combustion at 850 °C is feasible for the partial evaporation of all semivolatiles and volatiles except for Zn. At 1100 °C, Zn was also evaporated. All nonvolatiles remained entirely in the solid phase.

The addition of KCl changed the evaporation temperature for some elements. The order slightly changed, with S entering the gas phase after As, and Cu before Ni, with Zn before Cr. Except from Zn, heavy metals evaporated similarly to the addition of KCl with the addition of MgCl_2_ or CaCl_2_. Fraissler et al. suggested that Cr, Cu, Ni, Pb, and Zn tend to form gaseous Cl compounds, if Cl is added to SSA.^30^ According to Belevi and Langmeier, the formation of volatile chlorides is the reason for the lower evaporation temperature.^34^ According to our calculations and contrary to literature,^30,34^ the temperature of the complete evaporation of Zn increased by 360 °C.

Table 4 compares the temperature when 99% of an element was present in the gas phase according to TEC of tSS, tSS+, and tSS with the addition of 4 wt % MgCl_2_ or CaCl_2_. With additive addition, the evaporation temperature of Ni, Cu, Zn, Pb decreased,^30^ with MgCl_2_ showing the most significant effect. Addition of KCl in tSS+ achieved a comparable decrease, with the difference of increasing the evaporation temperature of Zn. The temperature for the evaporation of As, Cd, Tl, and Hg remained nearly the same for all additives used.

**Table 4:** TEC on the Influence of Additives on the Evaporation Temperature in °C

additive	P	K	Fe	Cr	Ni	Cu	S	Zn	As	Pb	Cd	Tl	Hg
	2470	2470	2210	1540	1380	1300	1120	1080	680	650	470	200	20
KCl	+20	+20	–10	–10	–230	–210	–80	+430	–30	–90	+10	–10	+0
MgCl_2_	–40	–30	0	+0	–330	–230	–70	–50	+0	–120	+0	+0	+0
CaCl_2_	+40	–30	–10	+0	–290	–230	–70	–40	+0	–100	+0	+0	+0

TEC in this study examined the influence of Cl donors on the evaporation of heavy metals. The composition of the sewage sludge was kept constant, and only one chloride was added at a time. It should be noted that P and S also influence the evaporation of heavy metals,^26,74–76^ which becomes important when the composition of the sewage sludge changes.

### 3.2 Gas Composition and Performance of Combustion

Figure 3a compares the main gas components of the exhaust. The goal air-fuel equivalence ratio λ was set to 1.15. Due to the flue gas fluctuations, the mean component values differed from one experiment to another. Hence, the mean O_2_ concentration in the flue gas ranged from 2.25 to 2.82 vol %. This means a λ between 1.135 and 1.175. CO_2_ also varied because of the flue gas fluctuations.

**Figure 3:**
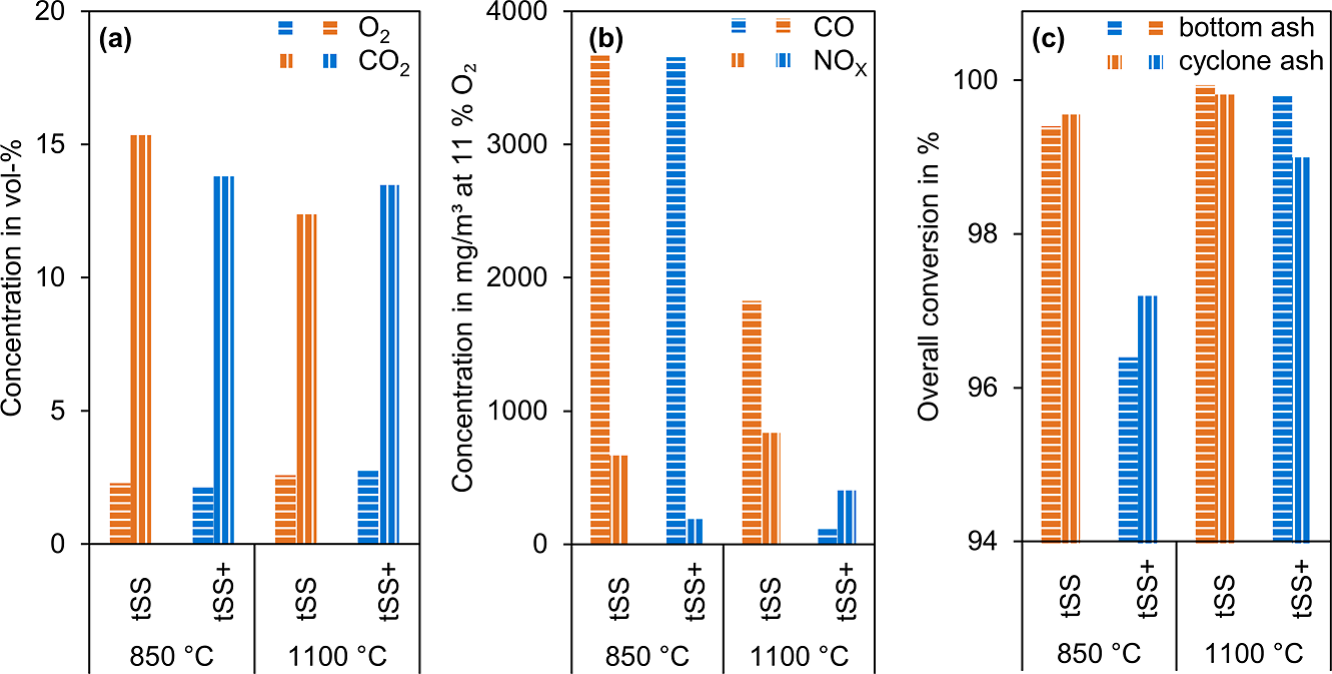
O_2_ and CO_2_ concentration (a), CO and NO_*x*_ concentration at 11°% O_2_ (b), and overall conversion (c) for PSSC of tSS and tSS+.

Figure 3b compares the CO and NO_*x*_ concentrations at 11% O_2_ calculated according to eq 8. CO decreased at a higher combustion temperatures due to better burn out of the solid fuel, with the CO emissions from the combustion with KCl as an additive displaying lower levels. NO_*x*_ emission levels increased at 1100 °C. Increasing temperature oxidizes larger amounts of fuel-bound N and forms thermal NO_*x*_ from N in the combustion air.^77,78^ However, they were reduced with tSS+ by 71.8% for the experiments at 850 °C and by 51.8% at 1100 °C compared to tSS. This reduction through K-additives was consistent with the literature, as K can reduce the formation of HCN and N_2_O as well as promote the reduction of NO_*x*_.^79,80^

Figure 3c shows the overall conversion according to eq 9 for the bottom ash and the cyclone ash. Similar values for both ashes confirmed the reliability of the determination. Overall conversion increased with the combustion temperature from 850 to 1100 °C. The addition of KCl led to lower conversion during combustion. The combustion of tSS+ at 850 °C resulted in a more incomplete conversion. Overall conversion was otherwise at 99% or above, which was nearly a complete combustion of all organic matter. During determination of the ash content, volatile components in the ash can evaporate in the MF and cause a distortion.^68^ The residence time when determining the ash content according to DIN 51719 was at least 4 h longer than in the EFCoRe.

### 3.3 Fate of P, K, S, and Heavy Metals

Figure 4 compares the content of P, K, S, and heavy metals of the fuel with the ash from the MF. The ash resulted from combustion at 550 °C according to DIN EN ISO 18122. Temperature in the MF was held for at least 4 h, which was a significantly longer residence time than in the EFCoRe. According to TEC, 550 °C was sufficient to evaporate Hg, Tl, Cd, and partly S from tSS and moreover some Pb from tSS+. Compared to experimental results, kinetic effects during a thermal process cannot be adequately taken into account in TEC and lead to deviations.^30^ Based on Fe as a tracer, the combustion of tSS in the MF was insufficient to evaporate significant amounts of P, K, Cr, Ni, Cu, Zn, As, Pb, and Cd. The only deviation from TEC was no evaporation of Cd and only partial evaporation of Tl. Based on Fe as a tracer, the combustion of tSS+ in the MF led to the partial evaporation of Cd and Pb. Overall, a prediction derived from TEC was accurate for the combustion in the MF at 550 °C.

**Figure 4:**
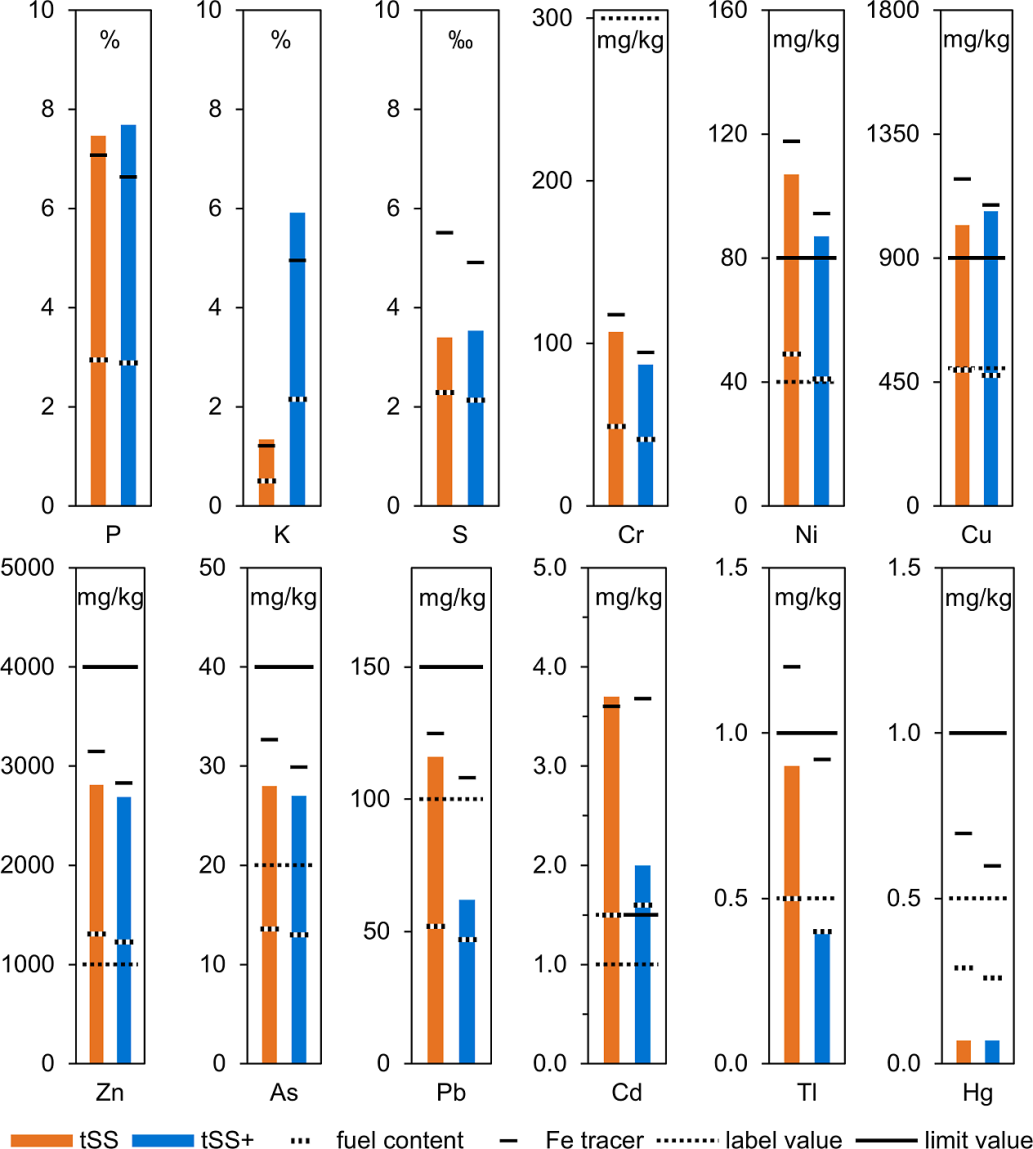
Content of P, K, S, and heavy metals of the ash from MF at 550 °C.

The remaining ash from combustion of tSS in the MF had a content of Ni, Cu, and Cd above the limit values according to DüMV. Zn, As, Pb, and Tl content was above the labeling threshold. Ash from the combustion of tSS+ in the MF complied with the labeling threshold of Pb and Tl.^62^ Therefore, the ash from combustion in the MF was unsuitable as a fertilizer. However, KCl significantly improved Pb, Cd, and Tl evaporation at 550 °C.

Even SSA, which complies with all DüMV limit values for heavy metals, often cannot be used directly as a fertilizer due of its poor P availability for plants.^7^ In the literature, an improvement^7^ and a deterioration^37^ of P bioavailability was observed when using KCl. For SSA from the MF at 550 °C, the solubility of P in neutral ammonium citrate was tested according to DIN EN 15957 and decreased from 97% for tSS to 72% for tSS+. To increase plant availability during combustion, more promising Cl donors should be used. According to the literature, MgCl_2_ and CaCl_2_ form compounds with P, which improve the plant availability of P from the SSA.^29,33,37,46,49^

In this study, discussion is focused on the bottom ash of the EFCoRe, leaving the reactor at a temperature of 300 to 400 °C. The cyclone separated the ash at 200 to 300 °C, where condensation of gaseous elements became more significant. The temperature in the filter for the fly ash was 60 to 70 °C, serving as a sink for volatile and condensable components of the ash.^19^ In some cases, the content in cyclone or fly ash is discussed to provide additional insights into the ash behavior. For each element’s content in the ash, the Fe tracer content *x*_*i*,Fe_ from eq 10 is provided. The Fe tracer content indicates an element’s content, when thermal treatment has no influence on the specific element. A lower content of the specific element indicates evaporation, while a higher content suggests enrichment.^73^

Figure 5a compares the P content of the fuel with the three types of ash. The P content in all samples was close to the content based on Fe as a tracer. The estimated content for P was between 94 and 98% in the bottom ash and between 89 and 94% in the cyclone ash. Fly ash contained slightly more P compared to the Fe tracer content, indicating a minor evaporation. Despite increasing temperature, PSSC of tSS and tSS+ had a minimal impact on the P content in each ash sample. An exact gravimetric determination of ash cannot be carried out in the EFCoRe due to diverse ash deposits. The amount of fly ash was in the single-digit percentage range compared to the total amount of ash. Therefore, the remaining ash contains sufficient amounts of P to be suitable for the recovery. According to AbklärV, at least 80% of the P must be recovered from SSA.^13^ According to literature^73^ and TEC, P behaves as a nonvolatile element in PSSC at the tested conditions.

**Figure 5:**
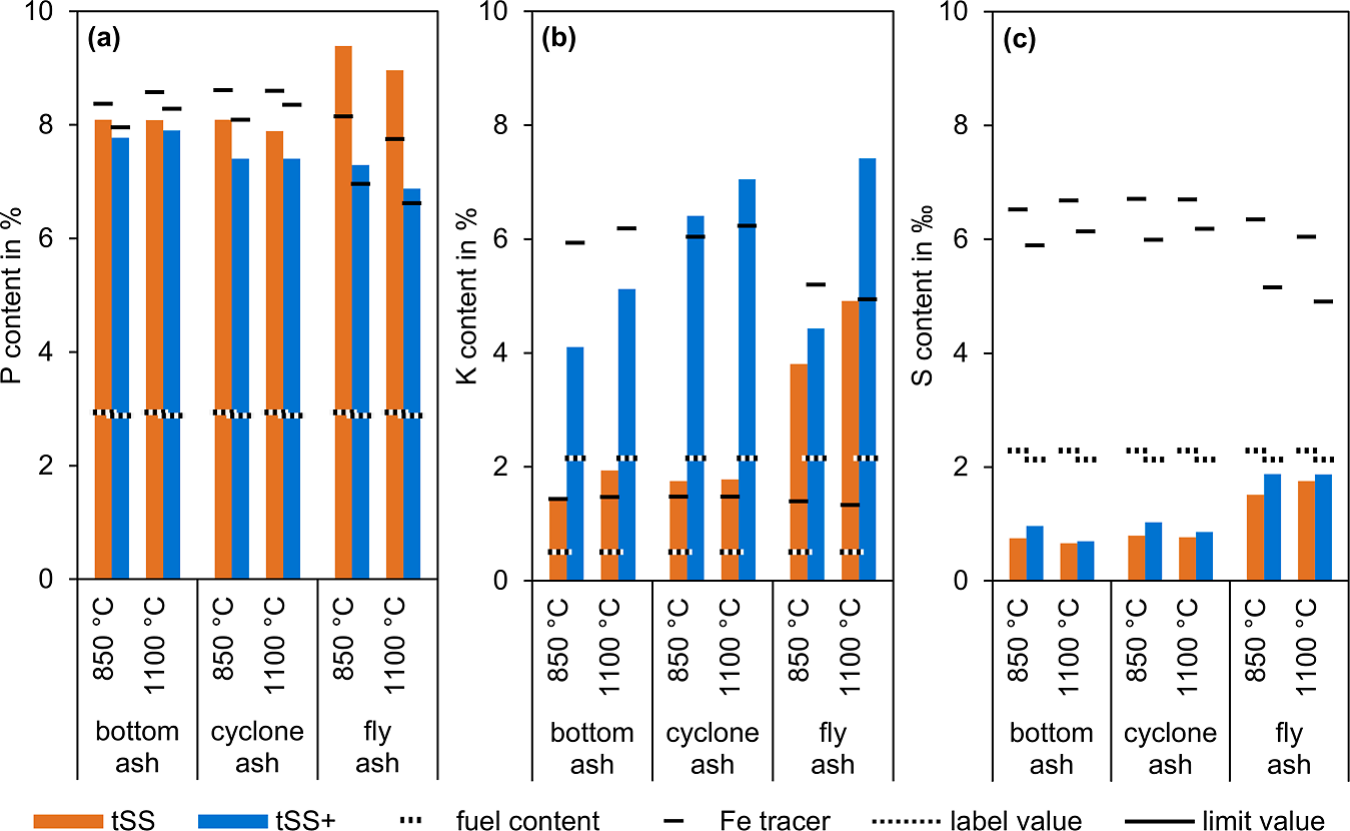
Content of P (a), K (b), and S (c) of the ash from PSSC.

Figure 5b compares the K content of the fuel with the three types of ash. For PSSC of tSS, K remained in the bottom ash, with a slightly elevated content in the fly ash. For PSSC of tSS+, the K content in the bottom ash decreased. The TEC shown in Figure 2 indicated K evaporation beginning at 1200 °C and across a broad temperature range. Increased evaporation of K during PSSC with KCl aligned with the TEC, except for the specific starting temperature.

Figure 5c compares the S content in the fuel with the three ash types. According to literature, S forms SO_2_ during combustion.^16^ Temperature had minimal influence on the S content of the ashes, but KCl in tSS+ decreased the evaporation of S. Overall, a significant share of S transitioned to the gas phase. The low S content in all ashes indicated that a subsequent flue gas treatment is mandatory to comply with limit values of the 17th BImSchV.^14^

Figure 6a compares the Cr content in the fuel with the three types of ash. Some components of the reactor consisted of stainless steel, which can contaminate the ash samples with Cr.^81^ Schnell and Quicker showed a highly unpredictable behavior in evaporation of Cr, where an increasing temperature led to an increasing content in the ash.^15^ This was attributed to the formation of solid oxide phases.^82^ Nonetheless, the Cr content for all ash samples was near or below the content based on the Fe tracer. Cr has a labeling threshold of 300 mg/kg in the DüMV, but no specific limit value.^62^ Since neither the Fe tracer nor any of the samples reached the labeling threshold, Cr was an unproblematic element, regarding the sewage sludge used as a fertilizer.

**Figure 6:**
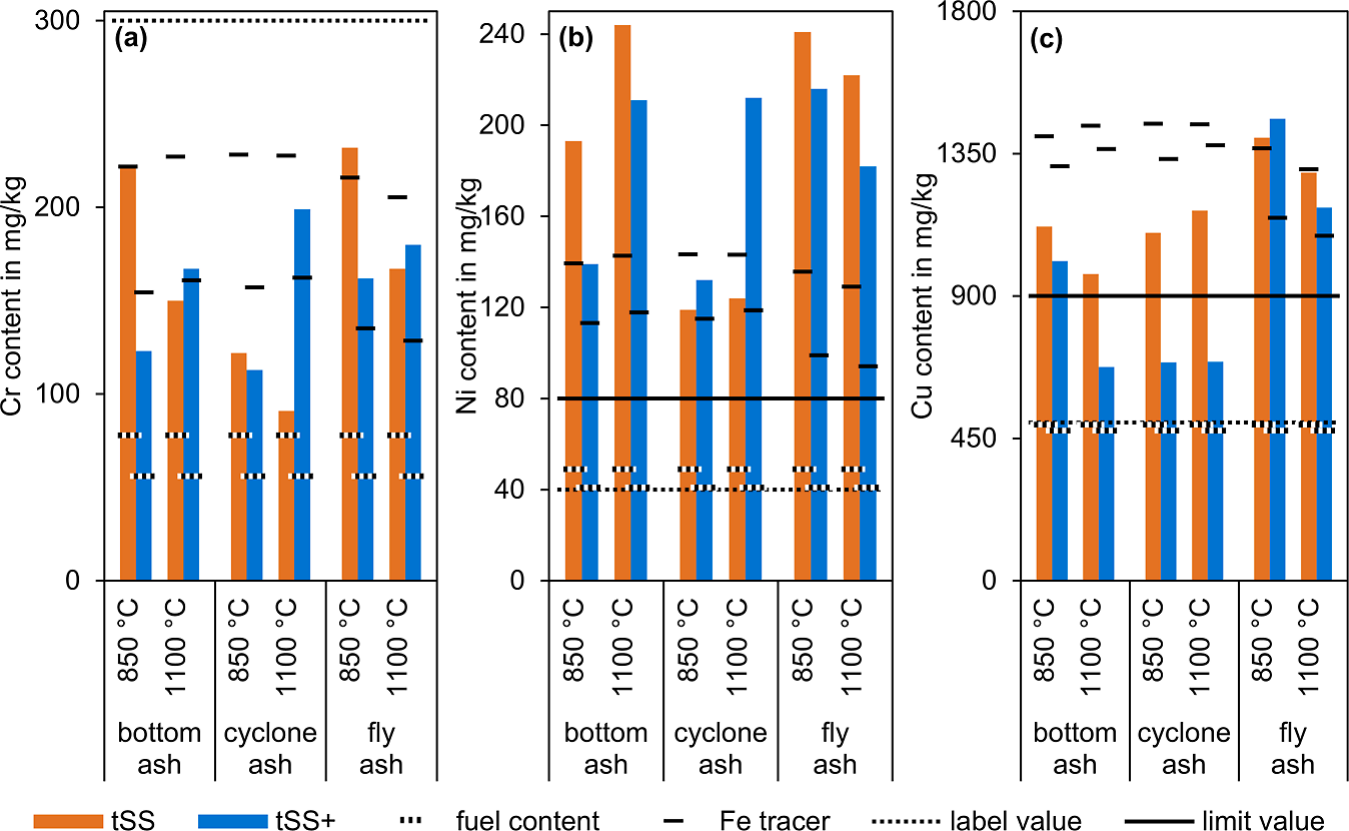
Content of Cr (a), Ni (b), and Cu (c) of the ash from PSSC.

Figure 6b compares the Ni content in the fuel with the three types of ash. Some components of the EFCoRe consisted of stainless steel. Similar to Cr, corrosion of stainless steel from the EFCoRe can affect the content of Ni in ash samples.^83^ In PSSC, some ash samples contained Ni significantly above the Fe tracer content, suggesting a contamination with excess Ni. However, ash from the MF from Figure 4 contained a Ni content slightly below the content based on Fe as a tracer. This confirmed contamination with excess Ni in PSSC. Ni is a problematic pollutant according to DüMV because all ash samples exceeded the limit of 80 mg/kg.^62^ Only the raw sewage sludge contained Ni below the limit value.

Figure 6c compares the Cu content in the fuel with the three types of ash. In PSSC, the higher temperature evaporated Cu from the bottom ash compared to combustion in the MF. According to the TEC, KCl in tSS+ lowered the temperature required for Cu evaporation. In PSSC of tSS+, KCl enhanced Cu evaporation at both temperatures. The DüMV sets a limit for Cu at 900 mg/kg,^62^ which most ash samples exceeded. Only PSSC at 1100 °C with tSS+ evaporated a sufficient amount Cu to comply with the limit value.

Figure 7a compares the Zn content in the fuel with the three types of ash. Compared to the Fe tracer content of Zn, PSSC achieved significant evaporation. According to TEC, KCl in tSS+ increased the temperature required for Zn evaporation. In the bottom ash from PSSC of tSS+, Zn evaporation was lower, aligning with TEC. The DüMV limit for Zn is 4000 mg/kg.^62^ The Fe tracer content for the bottom ash was close to the limit, suggesting potential for exceeding the limit. However, the Zn content in the bottom ash was close to the labeling threshold of 1000 mg/kg.^62^

**Figure 7:**
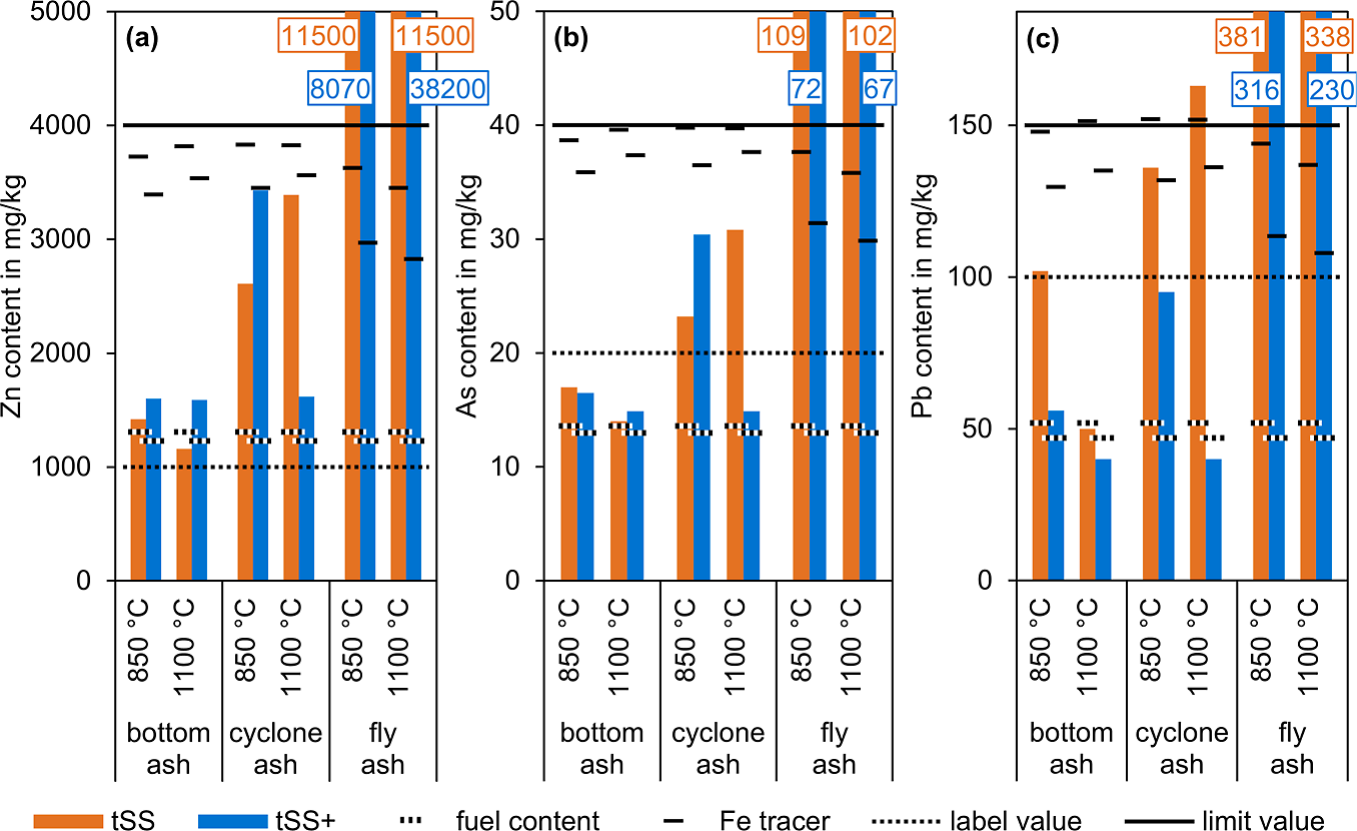
Content of Zn (a), As (b), and Pb (c) of the ash from PSSC.

Figure 7b compares the As content in the fuel with the three types of ash. In PSSC, As evaporated with increasing temperature but not with the addition of KCl in tSS+. According to TEC, the effect of KCl in tSS+ on As evaporation was minor. The estimated content for As based on Fe as a tracer was close to the limit according to DüMV of 40 mg/kg. However, PSSC reduced the As content in the bottom ash to below the labeling threshold of 20 mg/kg.^62^

Figure 7c compares the Pb content in the fuel with the three types of ash. According to TEC, the evaporation temperature for Pb was below the temperature in PSSC and the addition of KCl improved Pb evaporation. In PSSC, Pb evaporated as the temperature increased. Adding KCl in tSS+ enhanced the evaporation of Pb. The DüMV limit for Pb is 150 mg/kg,^62^ which was close to the content estimated by the Fe tracer. With PSSC at 850 °C, the bottom ash reached the labeling threshold of 100 mg/kg.^62^ Adding KCl or increasing the temperature to 1100 °C resulted in a Pb content of the bottom ash below the labeling threshold.

Figure 8a compares the Cd content in the fuel with the three types of ash. According to TEC, addition of KCl in tSS+ did not improve Cd evaporation. During PSSC, Cd evaporated with increasing temperature. However, adding KCl in tSS+ increased Cd reduction during PSSC. The DüMV limit for Cd is 1.5 mg/kg,^62^ and only the bottom ash from combustion of tSS+ or at 1100 °C complied with the limit value. Therefore, Cd was a challenging pollutant for thermal treatment of the sewage sludge used.

**Figure 8:**
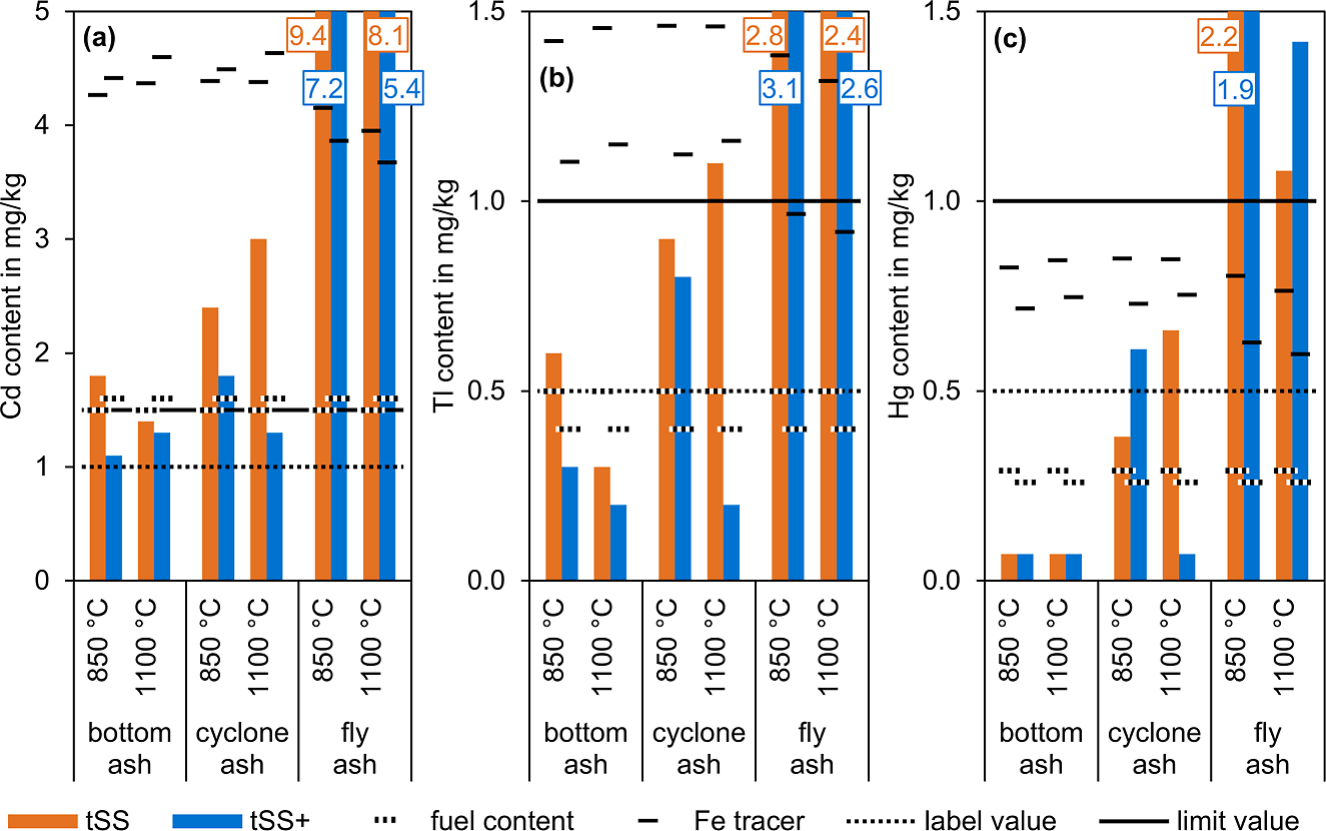
Content of Cd (a), Tl (b), and Hg (c) of the ash from PSSC.

Figure 8b compares the Tl content in the fuel with the three types of ash. According to TEC, the impact of KCl addition in tSS+ on Tl evaporation was minor. However, KCl in tSS+ significantly improved Tl evaporation during PSSC. The DüMV threshold for Tl is 1.0 mg/kg.^62^ According to the content based on Fe as a tracer, the ash from tSS and tSS+ had the potential to exceed the limit. For bottom ash, PSSC at 850 °C reduced the Tl content below the limit value. PSSC with tSS+ or at 1100 °C further reduced the Tl content of the bottom ash below the labeling threshold of 0.5 mg/kg.^62^ The combustion of tSS+ at 1100 °C reduced the Tl content to the detection limit of 0.2 mg/kg. Therefore, the content of Tl in this bottom ash could be lower than shown in Figure 8b.

Figure 8c compares the Hg content in the fuel with the three types of ash. After PSSC with tSS or tSS+, the Hg content of the bottom ash was below the labeling threshold of 0.5 mg/kg.^62^ PSSC reduced the Hg content to the detection limit of 0.07 mg/kg. Therefore, the content of Hg in all bottom ashes could be lower than shown in Figure 8c.

The comparison of results from the TEC with experimental measurements provided a coherent overall picture, although kinetic effects during the thermal process cannot be sufficiently accounted for in the calculations.^30^ The resulting limitations will be discussed in the summary.

According to the TEC, at 550 °C, only Hg, Tl, Cd, Pb, and S evaporated. These same elements evaporated in the MF experiments. At 850 °C, the TEC predicted that As and small amounts of Zn additionally evaporated. While the experiments confirmed the evaporation of As, the evaporation of Zn was more pronounced than expected. The TEC did not predict the minimal evaporation of Cu at 850 °C. At 1100 °C with the addition of KCl, the TEC predicted a significant effect on Cu, which was confirmed by the experiments. While the TEC was able to predict basic trends, it did not provide precise estimations. It was possible to show which elements would evaporate at a specific temperature, but the extent of the evaporation was not predicted.

In general, individual elements in the TEC typically transitioned to the gas phase within a narrow temperature range. In the experiments, the temperature range was much wider. The temperature at which evaporation was expected largely matched the experimental results. Even when an additive was introduced, the change in the evaporation temperature was reflected. Thus, the TEC alone could provide insights without the need for experimental measurements. The influence of individual additives, changes in the composition of the sewage sludge, or altered combustion temperatures can be estimated with minimal effort. However, the exact value of the evaporation of an element cannot be predicted by TEC. For an element with an evaporation temperature far below the combustion temperature, a significant removal from the ash can be expected.

## 4 Conclusions

Using TEC and experimental investigation in a MF and through PSSC, this study demonstrates how elevated temperatures and the addition of KCl in an entrained flow reactor produce SSA with low heavy metal content.

### 4.1 Theoretical considerations by TEC.

- P evaporation starts at 1500 °C, so the solid residue from the thermal treatment up to this temperature could serve as the raw material for P recovery.
- The addition of Cl in the form of KCl enhanced the evaporation of heavy metals during thermal treatment of sewage sludge comparably to more commonly used salts like MgCl_2_ and CaCl_2_.
- Compared with experimental results, the evaporation temperature from TEC provided an estimate with consistent order in general and consistent shifts due to the KCl addition.

### 4.2 Experimental Investigation in the MF

- Thermal treatment at 550 °C evaporated almost all Hg and a significant amount of Tl from the ash.
- By adding KCl, this effect was extended to the evaporation of Cd and Pb, whereas other heavy metals continued to not evaporate to a usable extent.

### 4.3 Experimental Investigation of PSSC

- In agreement with TEC, PSSC at 850 or 1100 °C kept P from the sewage sludge bound in the bottom ash, making it suitable as a potential raw material for fertilizer production.
- The smaller amount of fly ash from the cold section of the flue gas cleaning served as a sink for heavy metals.
- Despite the short residence time, PSSC at 850 °C evaporated significant amounts of Hg, Tl, Cd, Pb, As, Zn, and Cu. However, the reduction did not comply with all the limits according to DüMV.
- In contrast to stationary fluidized bed technology, PSSC allowed an elevated combustion temperature of 1100 °C, which improved the evaporation of heavy metals.
- For PSSC at 1100 °C and KCl addition, the bottom ash complied with all heavy metal limit values of the DüMV, except for Ni.

### 4.4 Limitations and Outlook

- The combustion setup used for PSSC contaminated the ash samples with additional Ni, covering a potential evaporation. According to TEC, the combination of KCl addition and the elevated temperature of 1100 °C evaporated Ni from the ash. However, an even higher temperature may be necessary for the effect to be sufficiently effective.
- The DüMV only specifies limit values for heavy metals, which is why possible changes in the environmental behavior of heavy metals are not considered in this study. The three-step sequential extraction procedure of the European Community Bureau of Reference or a X-ray absorption spectroscopy can help to clarify the migration of heavy metals into the environment.
- In an industrial application, hot gas filtration or cyclone separation is necessary to ensure that the largest amount of ash leaves the gas stream at a high temperature. If slagging operations can be implemented, a combustion temperature of up to 1500 °C would be useful, so that a larger amount of heavy metals evaporate.
- Since KCl did not contribute to improving the plant availability of P, it is recommended to switch to the chlorides MgCl_2_ or CaCl_2_, which are considered more promising according to the literature.

## Data Availability

All data are available within the manuscript.

## Abbreviations

AbfKlärV: German ordinance on the utilization of sewage sludge, sewage sludge mixture and sewage sludge compost
17th BImSchV: German ordinance on the incineration and co-incineration of waste
SSA: sewage sludge ash
DüMV: German fertilizer ordinance
PSSC: pulverized sewage sludge combustion
TEC: thermodynamic equilibrium calculations
EFCoRe: entrained flow combustion reactor
MF: muffle furnace
tSS: torrefied sewage sludge used
tSS+: torrefied sewage sludge used with added KCl.
